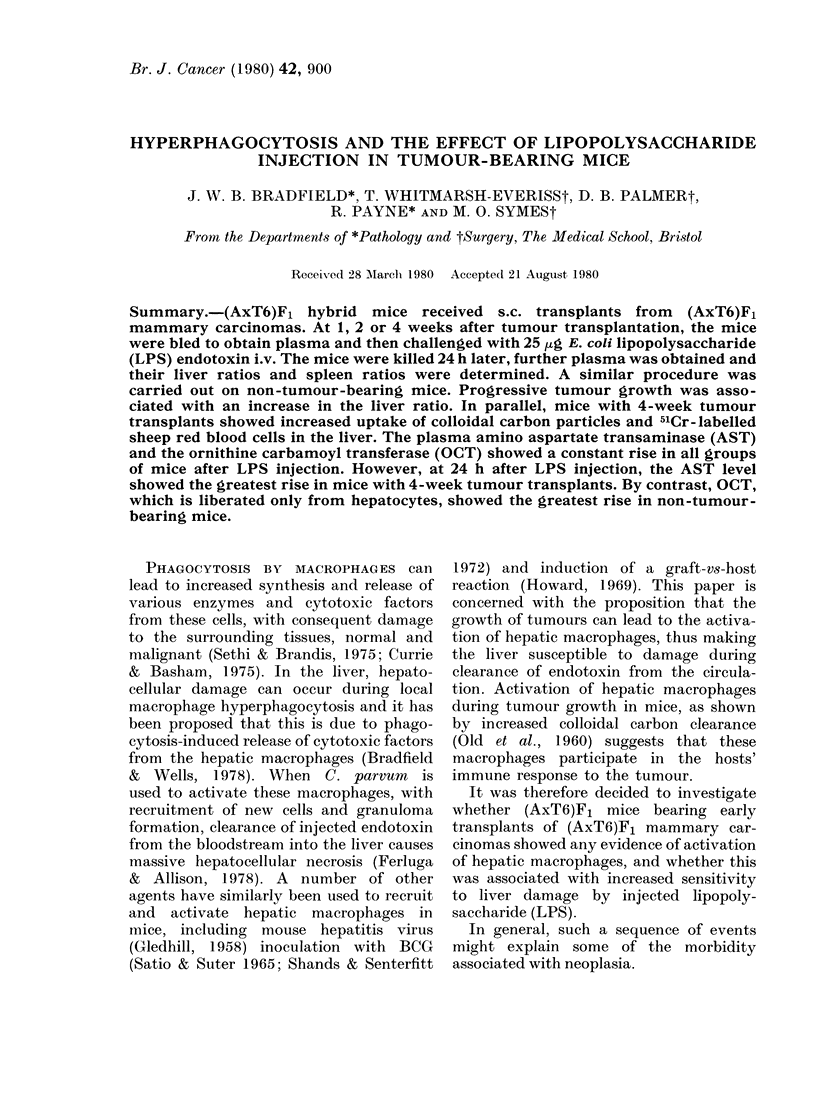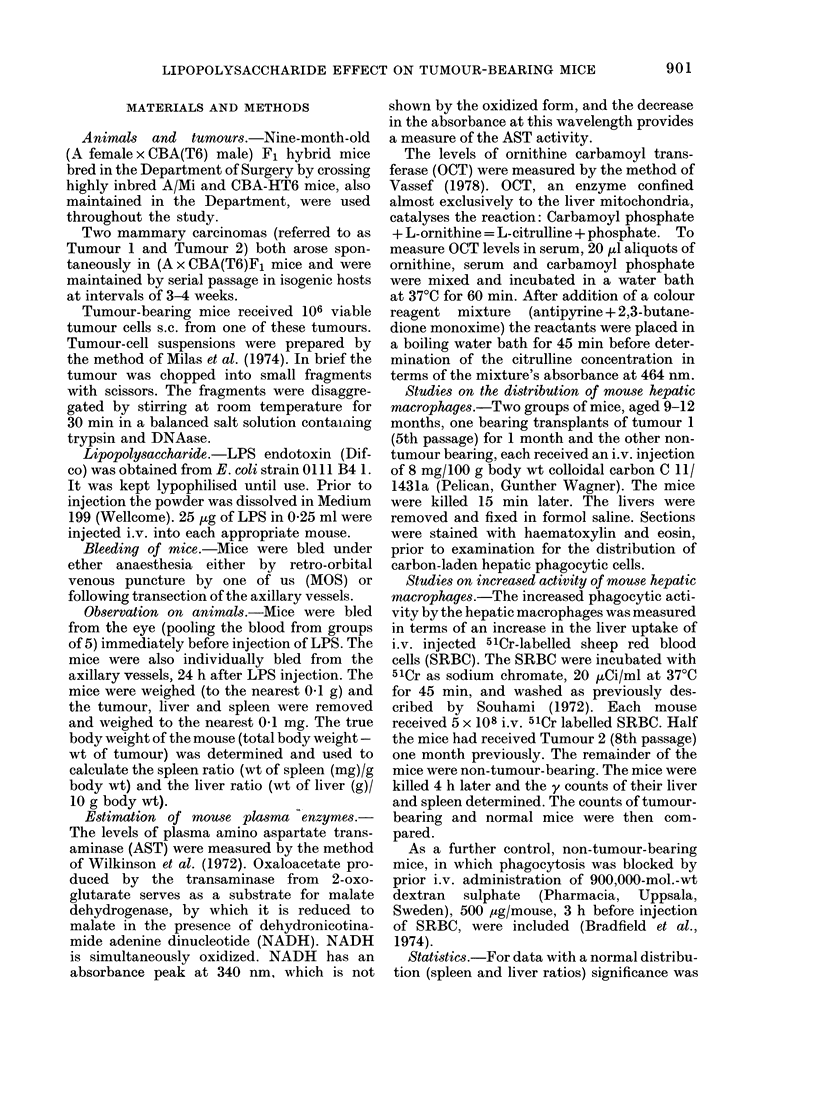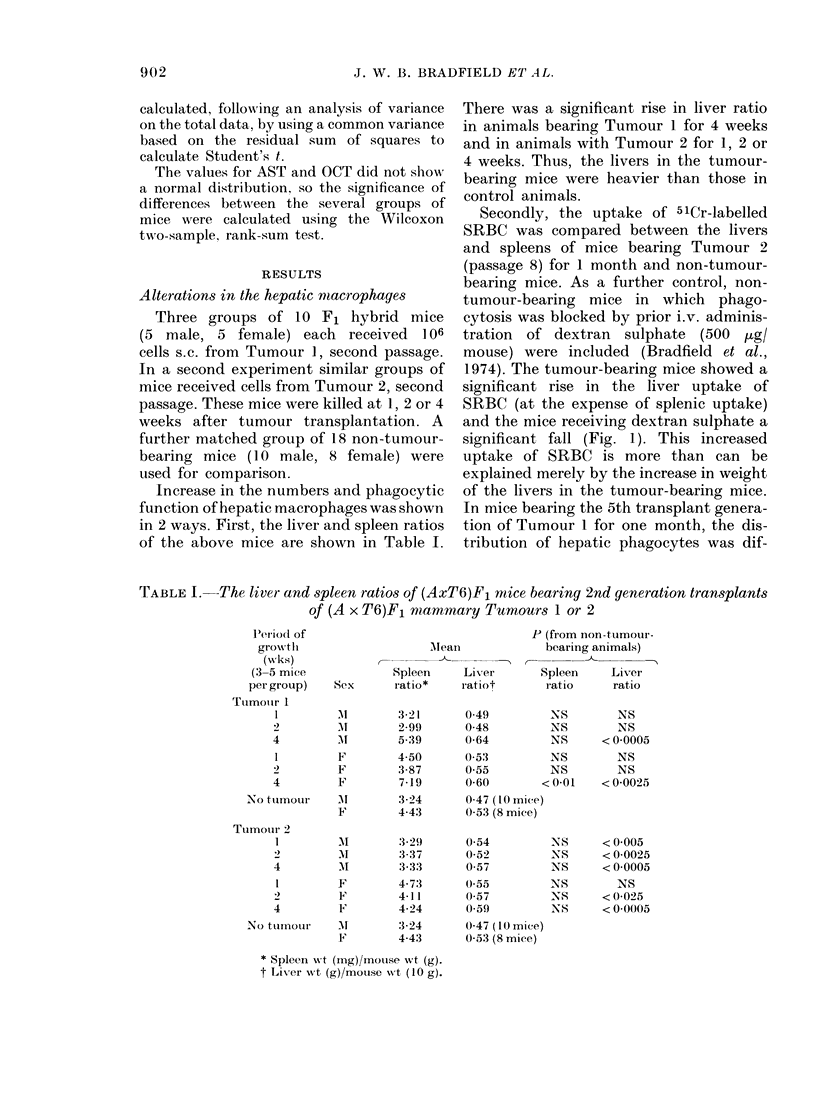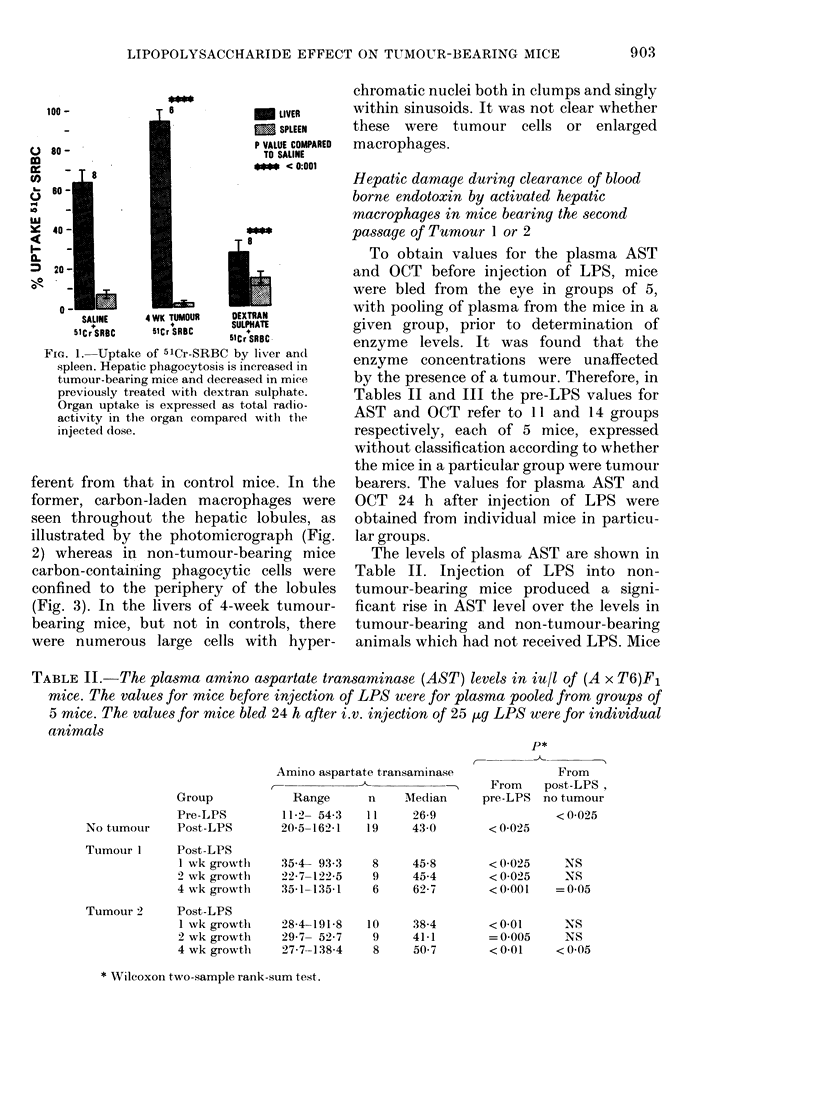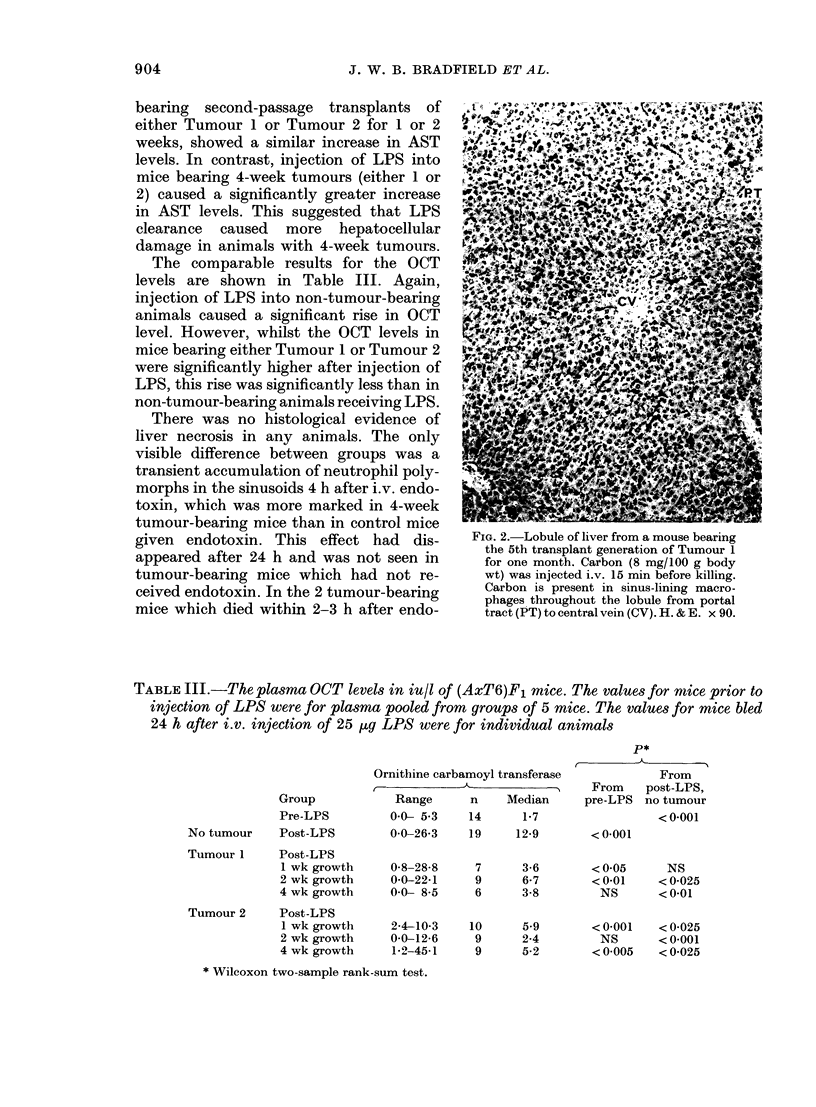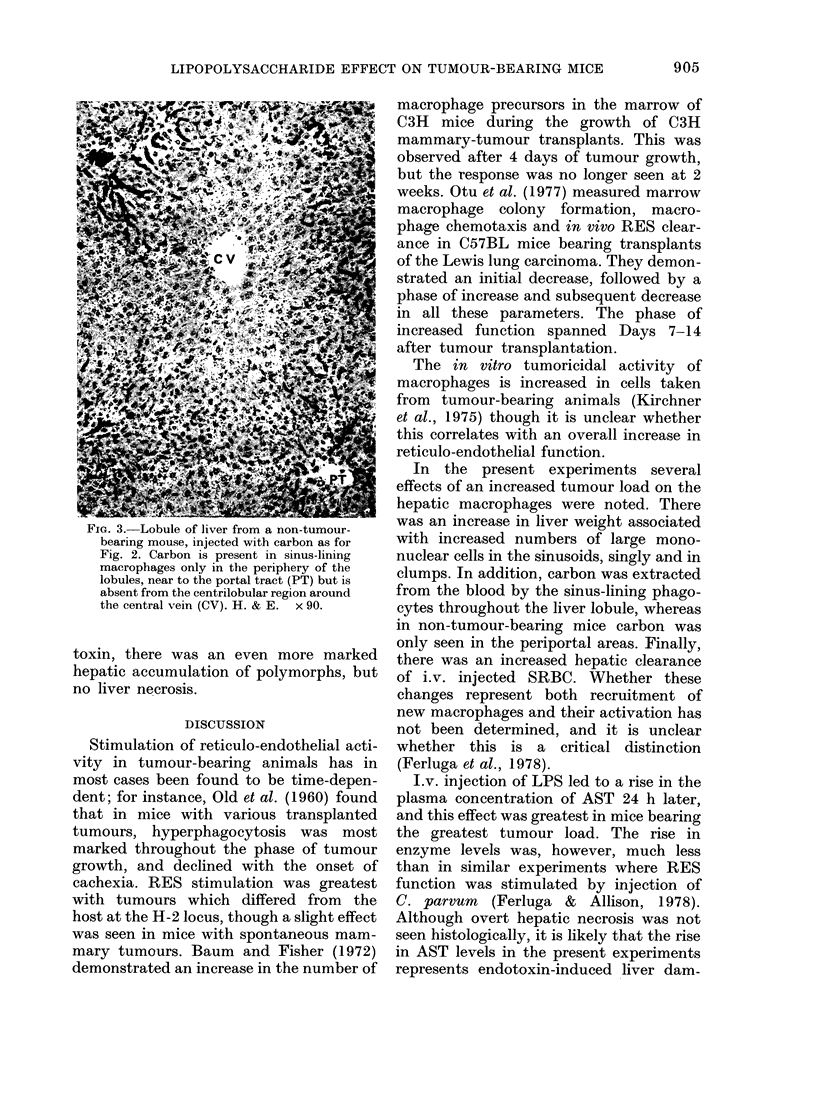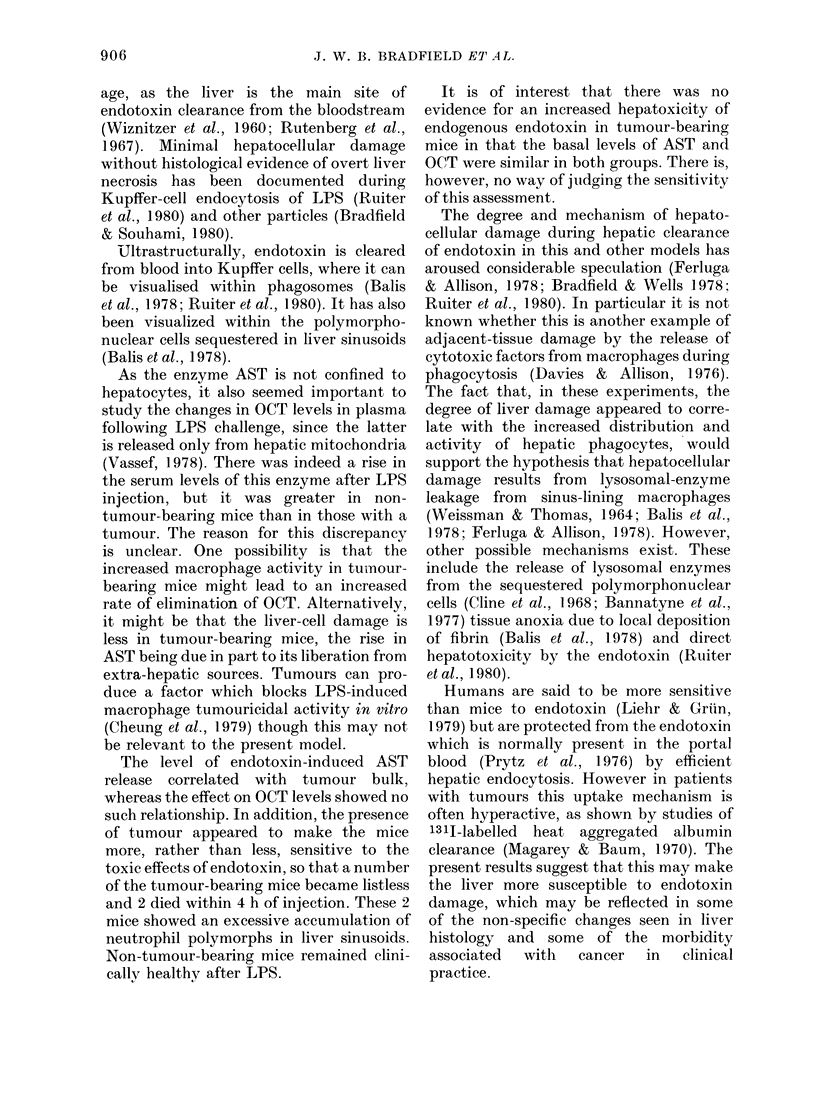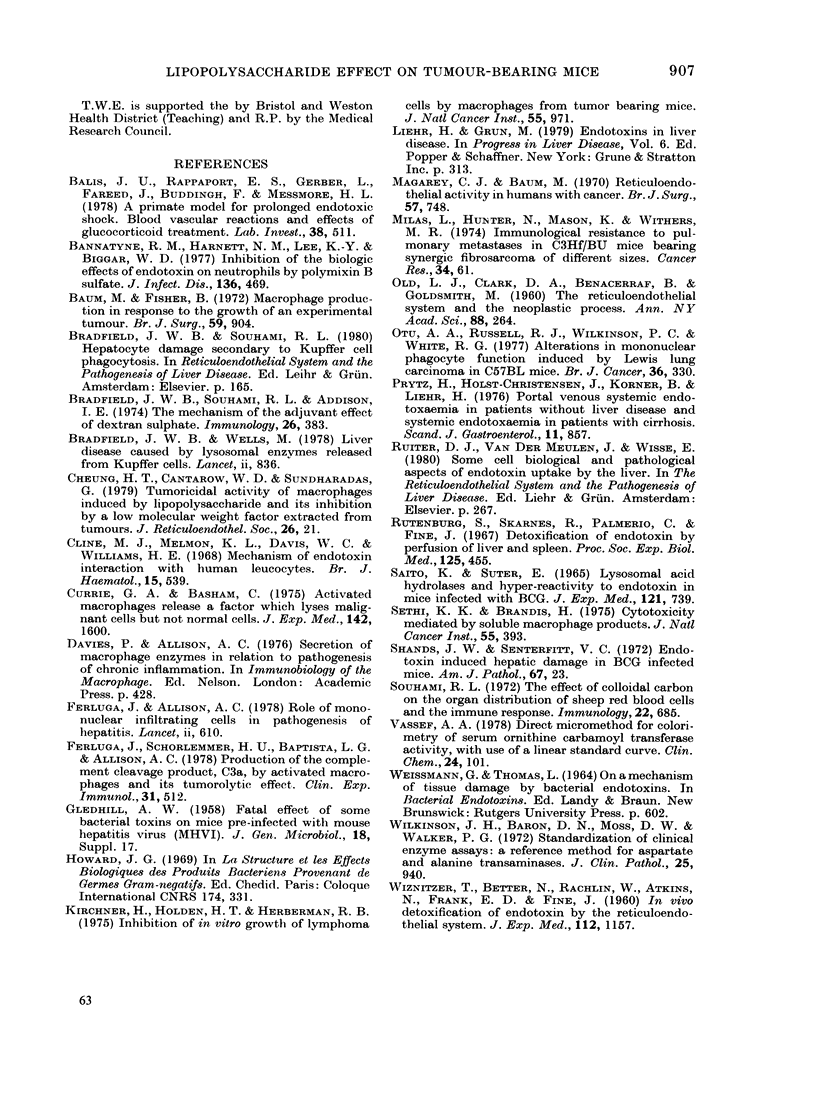# Hyperphagocytosis and the effect of lipopolysaccharide injection in tumour-bearing mice.

**DOI:** 10.1038/bjc.1980.338

**Published:** 1980-12

**Authors:** J. W. Bradfield, T. Whitmarsh-Everiss, D. B. Palmer, R. Payne, M. O. Symes

## Abstract

**Images:**


*Br. J. Cancer* (1980) **42**, 900

### HYPERPHAGOCYTOSIS AND THE EFFECT OF LIPOPOLYSACCHARIDE INJECTION IN TUMOUR-BEARING MICE

J. W. B. BRADFIELD*, T. WHITMARSH-EVERISS†, D. B. PALMER†, R. PAYNE* AND M. O. SYMES†

*From the Departments of *Pathology and †Surgery, The Medical School, Bristol*

Received 28 March 1980 Accepted 21 August 1980

**Summary.—(AxT6)$F_1$ hybrid mice received s.c. transplants from (AxT6)$F_1$ mammary carcinomas. At 1, 2 or 4 weeks after tumour transplantation, the mice were bled to obtain plasma and then challenged with 25 μg *E. coli* lipopolysaccharide (LPS) endotoxin i.v. The mice were killed 24 h later, further plasma was obtained and their liver ratios and spleen ratios were determined. A similar procedure was carried out on non-tumour-bearing mice. Progressive tumour growth was associated with an increase in the liver ratio. In parallel, mice with 4-week tumour transplants showed increased uptake of colloidal carbon particles and $^{51}$Cr-labelled sheep red blood cells in the liver. The plasma amino aspartate transaminase (AST) and the ornithine carbamoyl transferase (OCT) showed a constant rise in all groups of mice after LPS injection. However, at 24 h after LPS injection, the AST level showed the greatest rise in mice with 4-week tumour transplants. By contrast, OCT, which is liberated only from hepatocytes, showed the greatest rise in non-tumour-bearing mice.**

PHAGOCYTOSIS BY MACROPHAGES can lead to increased synthesis and release of various enzymes and cytotoxic factors from these cells, with consequent damage to the surrounding tissues, normal and malignant (Sethi & Brandis, 1975; Currie & Basham, 1975). In the liver, hepatocellular damage can occur during local macrophage hyperphagocytosis and it has been proposed that this is due to phagocytosis-induced release of cytotoxic factors from the hepatic macrophages (Bradfield & Wells, 1978). When *C. parvum* is used to activate these macrophages, with recruitment of new cells and granuloma formation, clearance of injected endotoxin from the bloodstream into the liver causes massive hepatocellular necrosis (Ferluga & Allison, 1978). A number of other agents have similarly been used to recruit and activate hepatic macrophages in mice, including mouse hepatitis virus (Gledhill, 1958) inoculation with BCG (Satio & Suter 1965; Shands & Senterfitt 1972) and induction of a graft-*vs*-host reaction (Howard, 1969). This paper is concerned with the proposition that the growth of tumours can lead to the activation of hepatic macrophages, thus making the liver susceptible to damage during clearance of endotoxin from the circulation. Activation of hepatic macrophages during tumour growth in mice, as shown by increased colloidal carbon clearance (Old *et al.*, 1960) suggests that these macrophages participate in the hosts' immune response to the tumour.

It was therefore decided to investigate whether (AxT6)$F_1$ mice bearing early transplants of (AxT6)$F_1$ mammary carcinomas showed any evidence of activation of hepatic macrophages, and whether this was associated with increased sensitivity to liver damage by injected lipopolysaccharide (LPS).

In general, such a sequence of events might explain some of the morbidity associated with neoplasia.

### MATERIALS AND METHODS

*Animals and tumours.*—Nine-month-old (A female × CBA(T6) male) $F_1$ hybrid mice bred in the Department of Surgery by crossing highly inbred A/Mi and CBA-HT6 mice, also maintained in the Department, were used throughout the study.

Two mammary carcinomas (referred to as Tumour 1 and Tumour 2) both arose spontaneously in (A × CBA(T6)$F_1$ mice and were maintained by serial passage in isogenic hosts at intervals of 3–4 weeks.

Tumour-bearing mice received $10^6$ viable tumour cells s.c. from one of these tumours. Tumour-cell suspensions were prepared by the method of Milas *et al.* (1974). In brief the tumour was chopped into small fragments with scissors. The fragments were disaggregated by stirring at room temperature for 30 min in a balanced salt solution containing trypsin and DNAase.

*Lipopolysaccharide.*—LPS endotoxin (Difco) was obtained from *E. coli* strain 0111 B4 1. It was kept lypophilised until use. Prior to injection the powder was dissolved in Medium 199 (Wellcome). 25 μg of LPS in 0·25 ml were injected i.v. into each appropriate mouse.

*Bleeding of mice.*—Mice were bled under ether anaesthesia either by retro-orbital venous puncture by one of us (MOS) or following transection of the axillary vessels.

*Observation on animals.*—Mice were bled from the eye (pooling the blood from groups of 5) immediately before injection of LPS. The mice were also individually bled from the axillary vessels, 24 h after LPS injection. The mice were weighed (to the nearest 0·1 g) and the tumour, liver and spleen were removed and weighed to the nearest 0·1 mg. The true body weight of the mouse (total body weight − wt of tumour) was determined and used to calculate the spleen ratio (wt of spleen (mg)/g body wt) and the liver ratio (wt of liver (g)/10 g body wt).

*Estimation of mouse plasma enzymes.*—The levels of plasma amino aspartate transaminase (AST) were measured by the method of Wilkinson *et al.* (1972). Oxaloacetate produced by the transaminase from 2-oxoglutarate serves as a substrate for malate dehydrogenase, by which it is reduced to malate in the presence of dehydronicotinamide adenine dinucleotide (NADH). NADH is simultaneously oxidized. NADH has an absorbance peak at 340 nm, which is not shown by the oxidized form, and the decrease in the absorbance at this wavelength provides a measure of the AST activity.

The levels of ornithine carbamoyl transferase (OCT) were measured by the method of Vassef (1978). OCT, an enzyme confined almost exclusively to the liver mitochondria, catalyses the reaction: Carbamoyl phosphate + L-ornithine = L-citrulline + phosphate. To measure OCT levels in serum, 20 μl aliquots of ornithine, serum and carbamoyl phosphate were mixed and incubated in a water bath at 37°C for 60 min. After addition of a colour reagent mixture (antipyrine + 2,3-butanedione monoxime) the reactants were placed in a boiling water bath for 45 min before determination of the citrulline concentration in terms of the mixture's absorbance at 464 nm.

*Studies on the distribution of mouse hepatic macrophages.*—Two groups of mice, aged 9–12 months, one bearing transplants of tumour 1 (5th passage) for 1 month and the other non-tumour bearing, each received an i.v. injection of 8 mg/100 g body wt colloidal carbon C 11/1431a (Pelican, Gunther Wagner). The mice were killed 15 min later. The livers were removed and fixed in formol saline. Sections were stained with haematoxylin and eosin, prior to examination for the distribution of carbon-laden hepatic phagocytic cells.

*Studies on increased activity of mouse hepatic macrophages.*—The increased phagocytic activity by the hepatic macrophages was measured in terms of an increase in the liver uptake of i.v. injected $^{51}Cr$-labelled sheep red blood cells (SRBC). The SRBC were incubated with $^{51}Cr$ as sodium chromate, 20 μCi/ml at 37°C for 45 min, and washed as previously described by Souhami (1972). Each mouse received $5 \times 10^8$ i.v. $^{51}Cr$ labelled SRBC. Half the mice had received Tumour 2 (8th passage) one month previously. The remainder of the mice were non-tumour-bearing. The mice were killed 4 h later and the γ counts of their liver and spleen determined. The counts of tumour-bearing and normal mice were then compared.

As a further control, non-tumour-bearing mice, in which phagocytosis was blocked by prior i.v. administration of 900,000-mol.-wt dextran sulphate (Pharmacia, Uppsala, Sweden), 500 μg/mouse, 3 h before injection of SRBC, were included (Bradfield *et al.*, 1974).

*Statistics.*—For data with a normal distribution (spleen and liver ratios) significance was calculated, following an analysis of variance on the total data, by using a common variance based on the residual sum of squares to calculate Student's *t*.

The values for AST and OCT did not show a normal distribution, so the significance of differences between the several groups of mice were calculated using the Wilcoxon two-sample, rank-sum test.

### RESULTS

### *Alterations in the hepatic macrophages*

Three groups of 10 $F_1$ hybrid mice (5 male, 5 female) each received $10^6$ cells s.c. from Tumour 1, second passage. In a second experiment similar groups of mice received cells from Tumour 2, second passage. These mice were killed at 1, 2 or 4 weeks after tumour transplantation. A further matched group of 18 non-tumour-bearing mice (10 male, 8 female) were used for comparison.

Increase in the numbers and phagocytic function of hepatic macrophages was shown in 2 ways. First, the liver and spleen ratios of the above mice are shown in Table I. There was a significant rise in liver ratio in animals bearing Tumour 1 for 4 weeks and in animals with Tumour 2 for 1, 2 or 4 weeks. Thus, the livers in the tumour-bearing mice were heavier than those in control animals.

Secondly, the uptake of $^{51}$Cr-labelled SRBC was compared between the livers and spleens of mice bearing Tumour 2 (passage 8) for 1 month and non-tumour-bearing mice. As a further control, non-tumour-bearing mice in which phagocytosis was blocked by prior i.v. administration of dextran sulphate (500 μg/mouse) were included (Bradfield *et al.*, 1974). The tumour-bearing mice showed a significant rise in the liver uptake of SRBC (at the expense of splenic uptake) and the mice receiving dextran sulphate a significant fall (Fig. 1). This increased uptake of SRBC is more than can be explained merely by the increase in weight of the livers in the tumour-bearing mice. In mice bearing the 5th transplant generation of Tumour 1 for one month, the distribution of hepatic phagocytes was dif-

TABLE I.—*The liver and spleen ratios of* $(AxT6)F_1$ *mice bearing 2nd generation transplants of* $(A \times T6)F_1$ *mammary Tumours 1 or 2*

| Period of growth (wks) (3–5 mice per group) | Sex | Mean | | *P* (from non-tumour-bearing animals) | |
|---|---|---|---|---|---|
| | | Spleen ratio* | Liver ratio† | Spleen ratio | Liver ratio |
| Tumour 1 | | | | | |
| 1 | M | 3·21 | 0·49 | NS | NS |
| 2 | M | 2·99 | 0·48 | NS | NS |
| 4 | M | 5·39 | 0·64 | NS | < 0·0005 |
| 1 | F | 4·50 | 0·53 | NS | NS |
| 2 | F | 3·87 | 0·55 | NS | NS |
| 4 | F | 7·19 | 0·60 | < 0·01 | < 0·0025 |
| No tumour | M | 3·24 | 0·47 (10 mice) | | |
| | F | 4·43 | 0·53 (8 mice) | | |
| Tumour 2 | | | | | |
| 1 | M | 3·29 | 0·54 | NS | < 0·005 |
| 2 | M | 3·37 | 0·52 | NS | < 0·0025 |
| 4 | M | 3·33 | 0·57 | NS | < 0·0005 |
| 1 | F | 4·73 | 0·55 | NS | NS |
| 2 | F | 4·11 | 0·57 | NS | < 0·025 |
| 4 | F | 4·24 | 0·59 | NS | < 0·0005 |
| No tumour | M | 3·24 | 0·47 (10 mice) | | |
| | F | 4·43 | 0·53 (8 mice) | | |

* Spleen wt (mg)/mouse wt (g).

† Liver wt (g)/mouse wt (10 g).

FIG. 1.—Uptake of $^{51}$Cr-SRBC by liver and spleen. Hepatic phagocytosis is increased in tumour-bearing mice and decreased in mice previously treated with dextran sulphate. Organ uptake is expressed as total radioactivity in the organ compared with the injected dose.

ferent from that in control mice. In the former, carbon-laden macrophages were seen throughout the hepatic lobules, as illustrated by the photomicrograph (Fig. 2) whereas in non-tumour-bearing mice carbon-containing phagocytic cells were confined to the periphery of the lobules (Fig. 3). In the livers of 4-week tumour-bearing mice, but not in controls, there were numerous large cells with hyperchromatic nuclei both in clumps and singly within sinusoids. It was not clear whether these were tumour cells or enlarged macrophages.

*Hepatic damage during clearance of blood borne endotoxin by activated hepatic macrophages in mice bearing the second passage of Tumour* 1 *or* 2

To obtain values for the plasma AST and OCT before injection of LPS, mice were bled from the eye in groups of 5, with pooling of plasma from the mice in a given group, prior to determination of enzyme levels. It was found that the enzyme concentrations were unaffected by the presence of a tumour. Therefore, in Tables II and III the pre-LPS values for AST and OCT refer to 11 and 14 groups respectively, each of 5 mice, expressed without classification according to whether the mice in a particular group were tumour bearers. The values for plasma AST and OCT 24 h after injection of LPS were obtained from individual mice in particular groups.

The levels of plasma AST are shown in Table II. Injection of LPS into non-tumour-bearing mice produced a significant rise in AST level over the levels in tumour-bearing and non-tumour-bearing animals which had not received LPS. Mice

TABLE II.—*The plasma amino aspartate transaminase* (*AST*) *levels in iu/l of* ($A \times T6)F_1$ *mice. The values for mice before injection of LPS were for plasma pooled from groups of* 5 *mice. The values for mice bled* 24 *h after i.v. injection of* 25 *μg LPS were for individual animals*

| | | Amino aspartate transaminase | | | P* | |
|---|---|---|---|---|---|---|
| | Group | Range | n | Median | From pre-LPS | From post-LPS, no tumour |
| | Pre-LPS | 11·2– 54·3 | 11 | 26·9 | | < 0·025 |
| No tumour | Post-LPS | 20·5–162·1 | 19 | 43·0 | < 0·025 | |
| Tumour 1 | Post-LPS | | | | | |
| | 1 wk growth | 35·4– 93·3 | 8 | 45·8 | < 0·025 | NS |
| | 2 wk growth | 22·7–122·5 | 9 | 45·4 | < 0·025 | NS |
| | 4 wk growth | 35·1–135·1 | 6 | 62·7 | < 0·001 | = 0·05 |
| Tumour 2 | Post-LPS | | | | | |
| | 1 wk growth | 28·4–191·8 | 10 | 38·4 | < 0·01 | NS |
| | 2 wk growth | 29·7– 52·7 | 9 | 41·1 | = 0·005 | NS |
| | 4 wk growth | 27·7–138·4 | 8 | 50·7 | < 0·01 | < 0·05 |

* Wilcoxon two-sample rank-sum test.

bearing second-passage transplants of either Tumour 1 or Tumour 2 for 1 or 2 weeks, showed a similar increase in AST levels. In contrast, injection of LPS into mice bearing 4-week tumours (either 1 or 2) caused a significantly greater increase in AST levels. This suggested that LPS clearance caused more hepatocellular damage in animals with 4-week tumours.

The comparable results for the OCT levels are shown in Table III. Again, injection of LPS into non-tumour-bearing animals caused a significant rise in OCT level. However, whilst the OCT levels in mice bearing either Tumour 1 or Tumour 2 were significantly higher after injection of LPS, this rise was significantly less than in non-tumour-bearing animals receiving LPS.

There was no histological evidence of liver necrosis in any animals. The only visible difference between groups was a transient accumulation of neutrophil polymorphs in the sinusoids 4 h after i.v. endotoxin, which was more marked in 4-week tumour-bearing mice than in control mice given endotoxin. This effect had disappeared after 24 h and was not seen in tumour-bearing mice which had not received endotoxin. In the 2 tumour-bearing mice which died within 2–3 h after endo-

FIG. 2.—Lobule of liver from a mouse bearing the 5th transplant generation of Tumour 1 for one month. Carbon (8 mg/100 g body wt) was injected i.v. 15 min before killing. Carbon is present in sinus-lining macrophages throughout the lobule from portal tract (PT) to central vein (CV). H. & E. × 90.

TABLE III.—*The plasma OCT levels in iu/l of* $(AxT6)F_1$ *mice. The values for mice prior to injection of LPS were for plasma pooled from groups of* 5 *mice. The values for mice bled* 24 *h after i.v. injection of* 25 *μg LPS were for individual animals*

| | | Ornithine carbamoyl transferase | | | P* | |
|---|---|---|---|---|---|---|
| | Group | Range | n | Median | From pre-LPS | From post-LPS, no tumour |
| | Pre-LPS | 0·0– 5·3 | 14 | 1·7 | | < 0·001 |
| No tumour | Post-LPS | 0·0–26·3 | 19 | 12·9 | < 0·001 | |
| Tumour 1 | Post-LPS | | | | | |
| | 1 wk growth | 0·8–28·8 | 7 | 3·6 | < 0·05 | NS |
| | 2 wk growth | 0·0–22·1 | 9 | 6·7 | < 0·01 | < 0·025 |
| | 4 wk growth | 0·0– 8·5 | 6 | 3·8 | NS | < 0·01 |
| Tumour 2 | Post-LPS | | | | | |
| | 1 wk growth | 2·4–10·3 | 10 | 5·9 | < 0·001 | < 0·025 |
| | 2 wk growth | 0·0–12·6 | 9 | 2·4 | NS | < 0·001 |
| | 4 wk growth | 1·2–45·1 | 9 | 5·2 | < 0·005 | < 0·025 |

* Wilcoxon two-sample rank-sum test.

FIG. 3.—Lobule of liver from a non-tumour-bearing mouse, injected with carbon as for Fig. 2. Carbon is present in sinus-lining macrophages only in the periphery of the lobules, near to the portal tract (PT) but is absent from the centrilobular region around the central vein (CV). H. & E. ×90.

toxin, there was an even more marked hepatic accumulation of polymorphs, but no liver necrosis.

### DISCUSSION

Stimulation of reticulo-endothelial activity in tumour-bearing animals has in most cases been found to be time-dependent; for instance, Old *et al.* (1960) found that in mice with various transplanted tumours, hyperphagocytosis was most marked throughout the phase of tumour growth, and declined with the onset of cachexia. RES stimulation was greatest with tumours which differed from the host at the H-2 locus, though a slight effect was seen in mice with spontaneous mammary tumours. Baum and Fisher (1972) demonstrated an increase in the number of macrophage precursors in the marrow of C3H mice during the growth of C3H mammary-tumour transplants. This was observed after 4 days of tumour growth, but the response was no longer seen at 2 weeks. Otu *et al.* (1977) measured marrow macrophage colony formation, macrophage chemotaxis and *in vivo* RES clearance in C57BL mice bearing transplants of the Lewis lung carcinoma. They demonstrated an initial decrease, followed by a phase of increase and subsequent decrease in all these parameters. The phase of increased function spanned Days 7–14 after tumour transplantation.

The *in vitro* tumoricidal activity of macrophages is increased in cells taken from tumour-bearing animals (Kirchner *et al.*, 1975) though it is unclear whether this correlates with an overall increase in reticulo-endothelial function.

In the present experiments several effects of an increased tumour load on the hepatic macrophages were noted. There was an increase in liver weight associated with increased numbers of large mononuclear cells in the sinusoids, singly and in clumps. In addition, carbon was extracted from the blood by the sinus-lining phagocytes throughout the liver lobule, whereas in non-tumour-bearing mice carbon was only seen in the periportal areas. Finally, there was an increased hepatic clearance of i.v. injected SRBC. Whether these changes represent both recruitment of new macrophages and their activation has not been determined, and it is unclear whether this is a critical distinction (Ferluga *et al.*, 1978).

I.v. injection of LPS led to a rise in the plasma concentration of AST 24 h later, and this effect was greatest in mice bearing the greatest tumour load. The rise in enzyme levels was, however, much less than in similar experiments where RES function was stimulated by injection of *C. parvum* (Ferluga & Allison, 1978). Although overt hepatic necrosis was not seen histologically, it is likely that the rise in AST levels in the present experiments represents endotoxin-induced liver damage, as the liver is the main site of endotoxin clearance from the bloodstream (Wiznitzer *et al.*, 1960; Rutenberg *et al.*, 1967). Minimal hepatocellular damage without histological evidence of overt liver necrosis has been documented during Kupffer-cell endocytosis of LPS (Ruiter *et al.*, 1980) and other particles (Bradfield & Souhami, 1980).

Ultrastructurally, endotoxin is cleared from blood into Kupffer cells, where it can be visualised within phagosomes (Balis *et al.*, 1978; Ruiter *et al.*, 1980). It has also been visualized within the polymorphonuclear cells sequestered in liver sinusoids (Balis *et al.*, 1978).

As the enzyme AST is not confined to hepatocytes, it also seemed important to study the changes in OCT levels in plasma following LPS challenge, since the latter is released only from hepatic mitochondria (Vassef, 1978). There was indeed a rise in the serum levels of this enzyme after LPS injection, but it was greater in non-tumour-bearing mice than in those with a tumour. The reason for this discrepancy is unclear. One possibility is that the increased macrophage activity in tumour-bearing mice might lead to an increased rate of elimination of OCT. Alternatively, it might be that the liver-cell damage is less in tumour-bearing mice, the rise in AST being due in part to its liberation from extra-hepatic sources. Tumours can produce a factor which blocks LPS-induced macrophage tumouricidal activity *in vitro* (Cheung *et al.*, 1979) though this may not be relevant to the present model.

The level of endotoxin-induced AST release correlated with tumour bulk, whereas the effect on OCT levels showed no such relationship. In addition, the presence of tumour appeared to make the mice more, rather than less, sensitive to the toxic effects of endotoxin, so that a number of the tumour-bearing mice became listless and 2 died within 4 h of injection. These 2 mice showed an excessive accumulation of neutrophil polymorphs in liver sinusoids. Non-tumour-bearing mice remained clinically healthy after LPS.

It is of interest that there was no evidence for an increased hepatoxicity of endogenous endotoxin in tumour-bearing mice in that the basal levels of AST and OCT were similar in both groups. There is, however, no way of judging the sensitivity of this assessment.

The degree and mechanism of hepatocellular damage during hepatic clearance of endotoxin in this and other models has aroused considerable speculation (Ferluga & Allison, 1978; Bradfield & Wells 1978; Ruiter *et al.*, 1980). In particular it is not known whether this is another example of adjacent-tissue damage by the release of cytotoxic factors from macrophages during phagocytosis (Davies & Allison, 1976). The fact that, in these experiments, the degree of liver damage appeared to correlate with the increased distribution and activity of hepatic phagocytes, would support the hypothesis that hepatocellular damage results from lysosomal-enzyme leakage from sinus-lining macrophages (Weissman & Thomas, 1964; Balis *et al.*, 1978; Ferluga & Allison, 1978). However, other possible mechanisms exist. These include the release of lysosomal enzymes from the sequestered polymorphonuclear cells (Cline *et al.*, 1968; Bannatyne *et al.*, 1977) tissue anoxia due to local deposition of fibrin (Balis *et al.*, 1978) and direct hepatotoxicity by the endotoxin (Ruiter *et al.*, 1980).

Humans are said to be more sensitive than mice to endotoxin (Liehr & Grün, 1979) but are protected from the endotoxin which is normally present in the portal blood (Prytz *et al.*, 1976) by efficient hepatic endocytosis. However in patients with tumours this uptake mechanism is often hyperactive, as shown by studies of $^{131}$I-labelled heat aggregated albumin clearance (Magarey & Baum, 1970). The present results suggest that this may make the liver more susceptible to endotoxin damage, which may be reflected in some of the non-specific changes seen in liver histology and some of the morbidity associated with cancer in clinical practice.

T.W.E. is supported the by Bristol and Weston Health District (Teaching) and R.P. by the Medical Research Council.

### REFERENCES

Balis, J. U., Rappaport, E. S., Gerber, L., Fareed, J., Buddingh, F. & Messmore, H. L. (1978) A primate model for prolonged endotoxic shock. Blood vascular reactions and effects of glucocorticoid treatment. *Lab. Invest.*, **38**, 511.

Bannatyne, R. M., Harnett, N. M., Lee, K.-Y. & Biggar, W. D. (1977) Inhibition of the biologic effects of endotoxin on neutrophils by polymixin B sulfate. *J. Infect. Dis.*, **136**, 469.

Baum, M. & Fisher, B. (1972) Macrophage production in response to the growth of an experimental tumour. *Br. J. Surg.*, **59**, 904.

Bradfield, J. W. B. & Souhami, R. L. (1980) Hepatocyte damage secondary to Kupffer cell phagocytosis. In *Reticuloendothelial System and the Pathogenesis of Liver Disease*. Ed. Leihr & Grün. Amsterdam: Elsevier. p. 165.

Bradfield, J. W. B., Souhami, R. L. & Addison, I. E. (1974) The mechanism of the adjuvant effect of dextran sulphate. *Immunology*, **26**, 383.

Bradfield, J. W. B. & Wells, M. (1978) Liver disease caused by lysosomal enzymes released from Kupffer cells. *Lancet*, ii, 836.

Cheung, H. T., Cantarow, W. D. & Sundharadas, G. (1979) Tumoricidal activity of macrophages induced by lipopolysaccharide and its inhibition by a low molecular weight factor extracted from tumours. *J. Reticuloendothel. Soc.*, **26**, 21.

Cline, M. J., Melmon, K. L., Davis, W. C. & Williams, H. E. (1968) Mechanism of endotoxin interaction with human leucocytes. *Br. J. Haematol.*, **15**, 539.

Currie, G. A. & Basham, C. (1975) Activated macrophages release a factor which lyses malignant cells but not normal cells. *J. Exp. Med.*, **142**, 1600.

Davies, P. & Allison, A. C. (1976) Secretion of macrophage enzymes in relation to pathogenesis of chronic inflammation. In *Immunobiology of the Macrophage*. Ed. Nelson. London: Academic Press. p. 428.

Ferluga, J. & Allison, A. C. (1978) Role of mononuclear infiltrating cells in pathogenesis of hepatitis. *Lancet*, ii, 610.

Ferluga, J., Schorlemmer, H. U., Baptista, L. G. & Allison, A. C. (1978) Production of the complement cleavage product, C3a, by activated macrophages and its tumorolytic effect. *Clin. Exp. Immunol.*, **31**, 512.

Gledhill, A. W. (1958) Fatal effect of some bacterial toxins on mice pre-infected with mouse hepatitis virus (MHVI). *J. Gen. Microbiol.*, **18**, Suppl. 17.

Howard, J. G. (1969) In *La Structure et les Effects Biologiques des Produits Bacteriens Provenant de Germes Gram-negatifs*. Ed. Chedid. Paris: Coloque International CNRS 174, 331.

Kirchner, H., Holden, H. T. & Herberman, R. B. (1975) Inhibition of *in vitro* growth of lymphoma cells by macrophages from tumor bearing mice. *J. Natl Cancer Inst.*, **55**, 971.

Liehr, H. & Grun, M. (1979) Endotoxins in liver disease. In *Progress in Liver Disease*, Vol. 6. Ed. Popper & Schaffner. New York: Grune & Stratton Inc. p. 313.

Magarey, C. J. & Baum, M. (1970) Reticuloendothelial activity in humans with cancer. *Br. J. Surg.*, **57**, 748.

Milas, L., Hunter, N., Mason, K. & Withers, M. R. (1974) Immunological resistance to pulmonary metastases in C3Hf/BU mice bearing synergic fibrosarcoma of different sizes. *Cancer Res.*, **34**, 61.

Old, L. J., Clark, D. A., Benacerraf, B. & Goldsmith, M. (1960) The reticuloendothelial system and the neoplastic process. *Ann. NY Acad. Sci.*, **88**, 264.

Otu, A. A., Russell, R. J., Wilkinson, P. C. & White, R. G. (1977) Alterations in mononuclear phagocyte function induced by Lewis lung carcinoma in C57BL mice. *Br. J. Cancer*, **36**, 330.

Prytz, H., Holst-Christensen, J., Korner, B. & Liehr, H. (1976) Portal venous systemic endotoxaemia in patients without liver disease and systemic endotoxaemia in patients with cirrhosis. *Scand. J. Gastroenterol.*, **11**, 857.

Ruiter, D. J., Van der Meulen, J. & Wisse, E. (1980) Some cell biological and pathological aspects of endotoxin uptake by the liver. In *The Reticuloendothelial System and the Pathogenesis of Liver Disease*. Ed. Liehr & Grün. Amsterdam: Elsevier. p. 267.

Rutenburg, S., Skarnes, R., Palmerio, C. & Fine, J. (1967) Detoxification of endotoxin by perfusion of liver and spleen. *Proc. Soc. Exp. Biol. Med.*, **125**, 455.

Saito, K. & Suter, E. (1965) Lysosomal acid hydrolases and hyper-reactivity to endotoxin in mice infected with BCG. *J. Exp. Med.*, **121**, 739.

Sethi, K. K. & Brandis, H. (1975) Cytotoxicity mediated by soluble macrophage products. *J. Natl Cancer Inst.*, **55**, 393.

Shands, J. W. & Senterfitt, V. C. (1972) Endotoxin induced hepatic damage in BCG infected mice. *Am. J. Pathol.*, **67**, 23.

Souhami, R. L. (1972) The effect of colloidal carbon on the organ distribution of sheep red blood cells and the immune response. *Immunology*, **22**, 685.

Vassef, A. A. (1978) Direct micromethod for colorimetry of serum ornithine carbamoyl transferase activity, with use of a linear standard curve. *Clin. Chem.*, **24**, 101.

Weissmann, G. & Thomas, L. (1964) On a mechanism of tissue damage by bacterial endotoxins. In *Bacterial Endotoxins*. Ed. Landy & Braun. New Brunswick: Rutgers University Press. p. 602.

Wilkinson, J. H., Baron, D. N., Moss, D. W. & Walker, P. G. (1972) Standardization of clinical enzyme assays: a reference method for aspartate and alanine transaminases. *J. Clin. Pathol.*, **25**, 940.

Wiznitzer, T., Better, N., Rachlin, W., Atkins, N., Frank, E. D. & Fine, J. (1960) *In vivo* detoxification of endotoxin by the reticuloendothelial system. *J. Exp. Med.*, **112**, 1157.